# Characterizing Oligomeric Hydroxyl Silicon Oils by MALDI-TOF MS With the Pyridine-Modified Matrix

**DOI:** 10.3389/fchem.2021.755174

**Published:** 2021-11-23

**Authors:** Xiaoxiao Zhang, Yan Wang, Yiqiu Hu, Cheng Guo, Chenghua Li, Kezhi Jiang

**Affiliations:** ^1^ College of Material, Chemistry and Chemical Engineering, Key Laboratory of Organosilicon Chemistry and Material Technology of Ministry of Education, Hangzhou Normal University, Hangzhou, China; ^2^ Cancer Institute (Key Laboratory of Cancer Prevention and Intervention, China National Ministry of Education), The Second Affiliated Hospital, Zhejiang University School of Medicine, Hangzhou, China

**Keywords:** silicon oil, MALDI-TOF, crystal structure of matrix, pyridine-modified DHB, ionization efficiency

## Abstract

Matrix-assisted laser desorption ionization time-of-flight mass spectrometry (MALDI-TOF) is a powerful technique for analysis of various polymers, but it is still very difficult to characterize silicone oil due to its poor ionization efficiency. In this work, oligomeric hydroxyl silicone oils were successfully characterized by MALDI-TOF, by using pyridine-modified 2,5-dihydroxylbenzoic acid (DHB) as the matrix. Furthermore, the mixed crystal of DHB and hydroxyl silicone oil was analyzed by scanning electron microscopy (SEM) and energy disperse spectroscopy (EDS), and the analytical results verified that modification with pyridine could remarkably improve the solubility of hydroxyl silicone oil in DHB, leading to the enhancement of its ionization efficiency in MALDI. The analysis of the MS spectra of a series of hydroxyl silicone oils indicated that they tended to be ionized by the attachment with Na^+^, and the average molecular weight and the degree of polymerization were measured for several oligomeric hydroxyl silicon oils.

## Introduction

**FIGURE 1:**
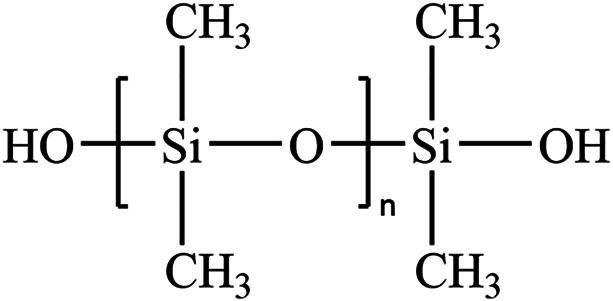
Structure of hydroxyl silicone oil.

Hydroxyl silicone oil refers to a linear polysiloxane with the Si–O–Si bond as the main chain and the silicon hydroxyl as the end group in the structure (Figure 1). It is usually maintained in the liquid state at ambient temperature. Owing to its plentiful merits, such as electric insulation, anti-high and -low temperatures, chemical inertia, physiological inertia, low surface tension, and water-repellent and moisture-resistant performance, silicone oil and its derivative products have been extensively applied to electricity, light industry, construction, and other fields (Chen et al., 2009; Mei et al., 2014; Aziz et al., 2018; Zhang et al., 2020). Currently, infrared (IR) spectroscopy, nuclear magnetic resonance (NMR), gel permeation chromatography (GPC), supercritical fluid chromatography (SFC), and matrix-assisted laser desorption ionization time-of-flight mass spectrometry (MALDI-TOF) are the powerful techniques to characterize silicone oil (Semchyschyn et al., 2000; Chmelik et al., 2001; Ren et al., 2019; Liu et al., 2021). GPC is a popular technique to determine the average molecular weight of polymers, but it is not suitable for the analysis of oligomeric hydroxyl silicone oils (Montaudo et al., 1995). Thus, it is essential to develop an alternative method for the characterization of the average molecular weight of oligomeric hydroxyl silicone oils.

MALDI-TOF has been widely applied for characterizing peptides, proteins, oligomers, and polymers since its invention in the 1980s, due to its high sensitivity and convenient operation (Karas et al., 1988; Tanaka et al., 1988; Li et al., 2019). The MALDI-TOF analysis can provide many important information of polymers, including the repeating unit, the molecular weight distribution, and the end group (Yalcin et al., 1997; Hanton et al., 2000; Pasch et al., 2000; Bauer et al., 2002; Peacock et al., 2004). Thus, it has become an important technique to characterize oligomers or polymers (Scrivens et al., 2000; Chmelik et al., 2001; Hanton, 2001). However, silicone oil belongs to a non-polar polymer, and it is very difficult to be ionized in the MALDI source (Mautjana et al., 2012).

Interestingly, it has been reported that the addition of some organic bases could improve the homogeneous distribution of the analyte in the traditional matrix and increase the dot-to-dot reproducibility in MALDI-TOF analysis (Snovida et al., 2008). In this work, oligomeric hydroxyl silicone oils were characterized by MALDI-TOF with the pyridine-modified 2,5-dihydroxylbenzoic acid (DHB) as the matrix, and the corresponding solid crystals were analyzed by scanning electron microscopy (SEM) and energy disperse spectroscopy (EDS) in order to probe the intrinsic mechanism on the improvement of the ionization efficiency originating from the modification of the matrix with pyridine.

## Experiment

### Reagents and Materials

Hydroxyl silicone oils with different viscosities were purchased from Qingdao Fenghong Chemical Co., Ltd. (Shandong, China). High-performance liquid chromatography (HPLC)-grade methanol (MeOH) was purchased from Sigma-Aldrich (St. Louis, MO, United States). HPLC-grade tetrahydrofuran (THF) was purchased from Merck Millipore (Billerica, MA, United States). 2,5-Dihydroxylbenzoic acid (DHB) was purchased from Shanghai Macklin Biochemical Co., Ltd. (Shanghai, China). Sodium acetate (NaAc) and pyridine were purchased from Sinopharm (China). The water used in all experiments was prepared in a Milli-Q water purification system with a resistivity ≥18.2 MΩ cm^−1^.

### Instruments

The microflex MALDI-TOF system was produced by Bruker Corporation (Germany). The BS110S precision balance was produced by Sartorius (Germany). The YM-080S Ultrasonic Cleaner was manufactured by Fang Ao Microelectronics Co., Ltd. (Shenzhen, Guangdong, China). The Sigma 500 scanning electron microscope (SEM) was produced by Zeiss (Germany). The energy disperse spectroscopy (EDS) system was produced by EDAX (United States).

### Experimental Procedure

DHB was weighted and dissolved in THF to prepare a 100 mg/mL solution. 50 μL pyridine solution was added into 1.0 mL DHB solution to prepare a solution of pyridine-modified DHB. The cationization reagent (NaAc) was weighted and dissolved in MeOH/H_2_O (50:1, V:V) to prepare a 100 mM solution. Hydroxyl silicone oils were weighted separately and dissolved in THF to prepare a 1 mg/mL solution. The mixed solution was prepared by mixing the above solutions according to oligomer/matrix/NaAc (or THF) ratio (1:5:1, V/V/V), and the dissolving process was assisted by ultrasound.

In MALDI-TOF experiments, 1.0 μL mixed solution was dried on a stainless steel target at room temperature for MALDI-TOF analysis. The operating parameters of MALDI-TOF were as follows: the nitrogen laser wavelength was 337 nm and the laser pulse width was 3 ns. In the direct radiation mode, the acceleration voltage was 20.0 kV and the reflection voltage was 23.0 kV. A single scan of the mass spectrum signal was added up to 100 times.

In SEM and EDS experiments, 10.0 μL mixed solution was dropped on a tin foil to dry, and the formed dry point was sprayed with platinum to enhance its electrical conductivity. Then, the dry point was subjected to SEM and EDS analysis. The SEM analysis was carried out at the testing voltage of 3 kV under the vacuum of 5.4 × 10^−8^ Pa. The EDS analysis was carried out at the testing voltage of 10 kV.

## Results and Discussion

### Effect of the Modified Matrix on the Ionization Efficiency

**FIGURE 2:**
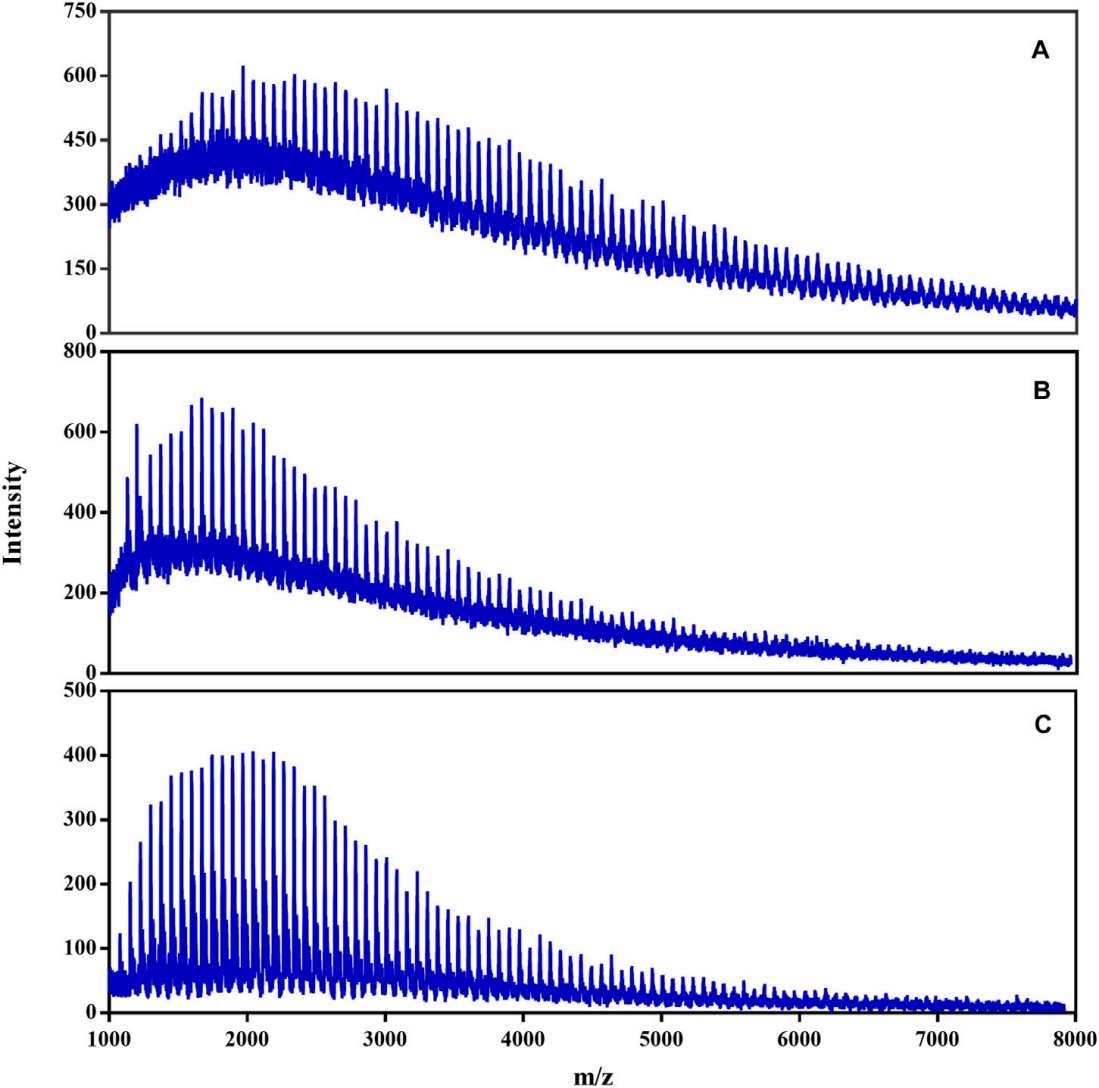
MALDI-TOF MS of 30 cP hydroxyl silicone oil with the different matrix: A) DHB, B) pyridine-modified DHB, C) pyridine-modified DHB with the addition of NaAc.

The 30 cP hydroxyl silicone oil was selected as a model for the MALDI-TOF analysis to investigate the effect of matrix on the ionization efficiency. As shown in Figure 2, the MS showed a series of equidistant peaks and an approximate *t*-distribution in the intensity of the MS signals, indicating a classical MS of the polymer. The mass gap of 74 Da for the neighboring peaks in the MS indicated the signal of silicone oil with the repeating unit of (SiOMe_2_). With the pure DHB as the MALDI matrix (Figure 2A), the intensity of the silicone oil signal was about 600 at 1800 Da, while that of the corresponding noise reached 400, indicating a bad signal-to-noise ratio (S/N). At the same time, the addition of ionization agent (NaAc) into the DHB matrix could not significantly improve the ionization efficiency of the hydroxyl silicone oils in the MALDI-TOF MS (Supplementary Figure S1). To be interesting, the corresponding S/N increased about two times with the pyridine-modified DHB as the matrix (Figure 2B). What’s more exciting, the noise intensity dropped to about 50, and thus, the corresponding S/N increased to 8 with the addition of some NaAc into the pyridine-modified DHB matrix. Thus, the MALDI-TOF MS was competent for structure characterization of hydroxyl silicone oils.

Similar results were obtained for MALDI-TOF analysis of the 50 cP and the 150 cP hydroxyl silicone oils (Supplementary Figures S2 and S3). With modification of the matrix, an enough intensive signal was produced for the MALDI-TOF MS of hydroxyl silicone oils, and thus various structural information could be obtained from the MALDI-TOF analysis.

### SEM and EDS Characterizing the Mixed Crystal of Matrix and Analyte

**FIGURE 3:**
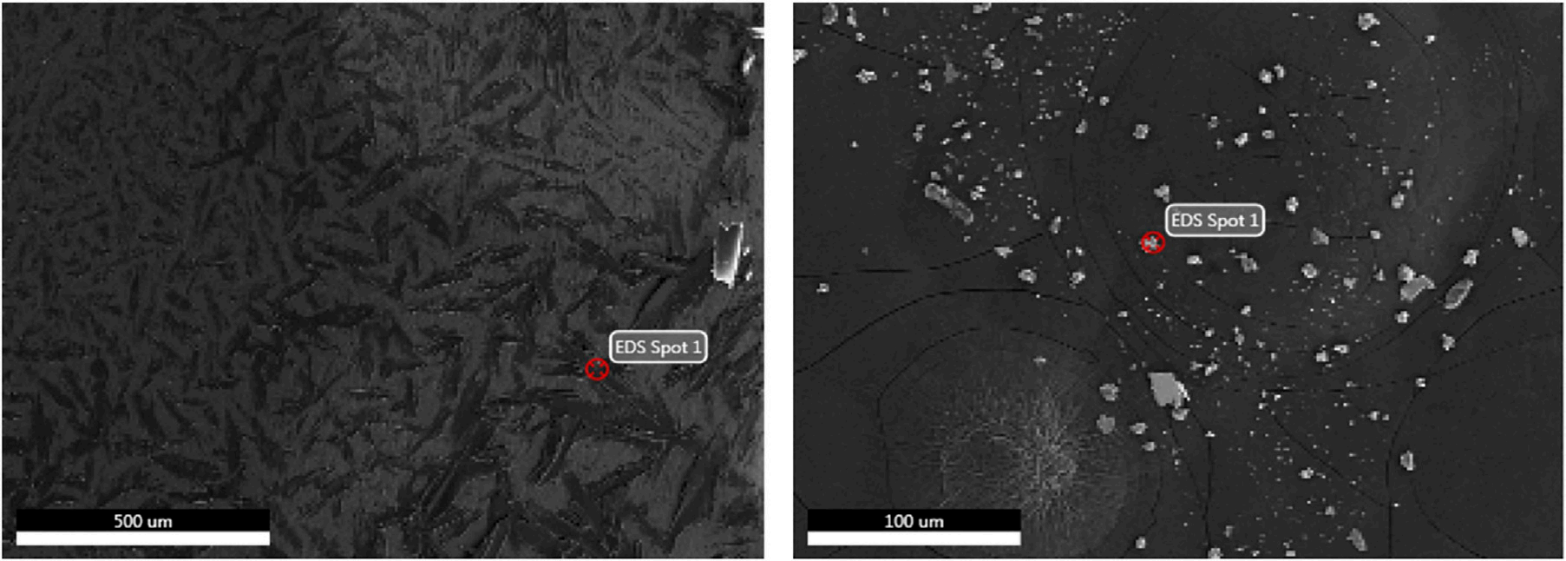
SEM images of the schistose crystal (left) and scattered particles (right) in the mixed crystal of DHB and 30 cP hydroxyl silicone oil.

**FIGURE 4:**
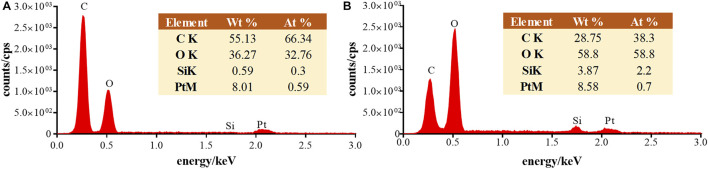
EDS images of the schistose crystal **(A)** and the particle **(B)** in mixed crystal of DHB and 30 cP hydroxyl silicone oil.

In order to further investigate the effect of matrix on the ionization efficiency, the mixed crystal of matrix and analyte was characterized by SEM and EDS. Figure 3 shows the SEM of the mixed crystal of DHB and 30 cP hydroxyl silicone oil, in which there were full of the schistose crystal with the irregular surface and scattered particles with different diameters at the macro-scale level of 100 μm. EDS analysis of the schistose crystals (Figure 4A) showed the main elements of C and O, indicating the identity of compound DHB. In contrast, there was significantly more content of both O and Si in the EDS of the particle (Figure 4B), which was consistent with the identity of hydroxyl silicone oil. Thereby, the silicone oil was heterogeneously distributed in the DHB matrix.

**FIGURE 5:**
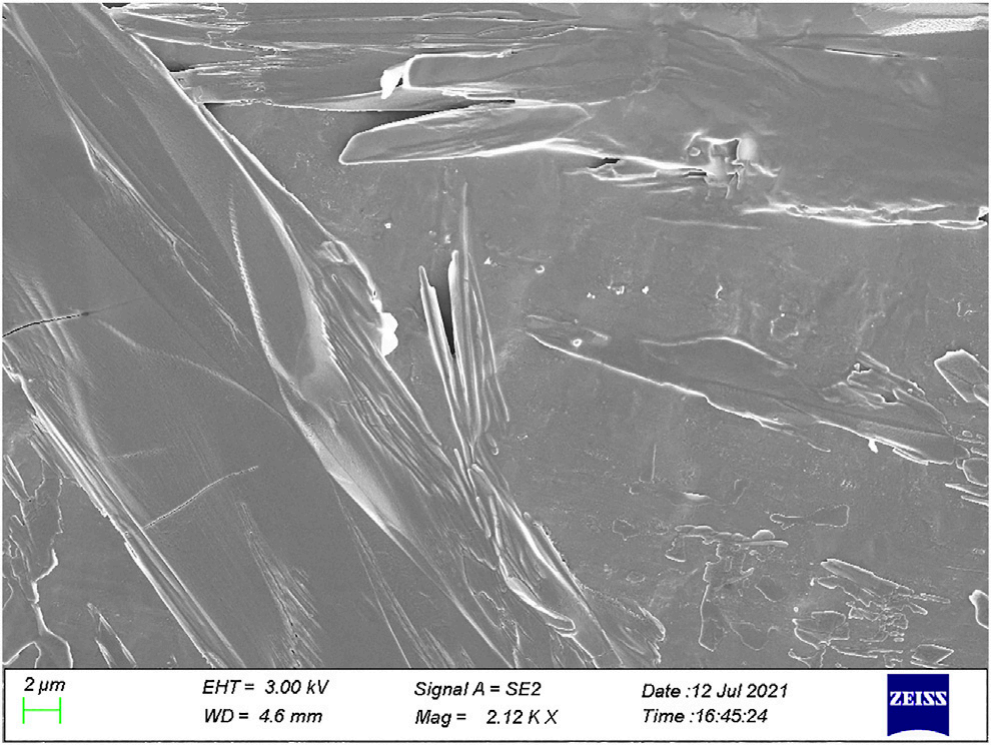
SEM image (at the 2 μm level) of the mixed crystal of DHB and 30 cP hydroxyl silicone oil.

Further magnification of the mixed crystal at a scale level of 2 μm resulted in many irregular tabular crystals with the obvious interface (Figure 5). The corresponding width was found at the μm-scale level. Similarly, the addition of NaAc into DHB did not significantly change the shape of the mixed crystal of matrix and analyte (Supplementary Figure S4). The above experimental results indicated that DHB had poor solubility with hydroxyl silicone oil, and thus, poor ionization efficiency was obtained for MALDI-TOF analysis of hydroxyl silicone oil with the pure DHB as the matrix.

**FIGURE 6:**
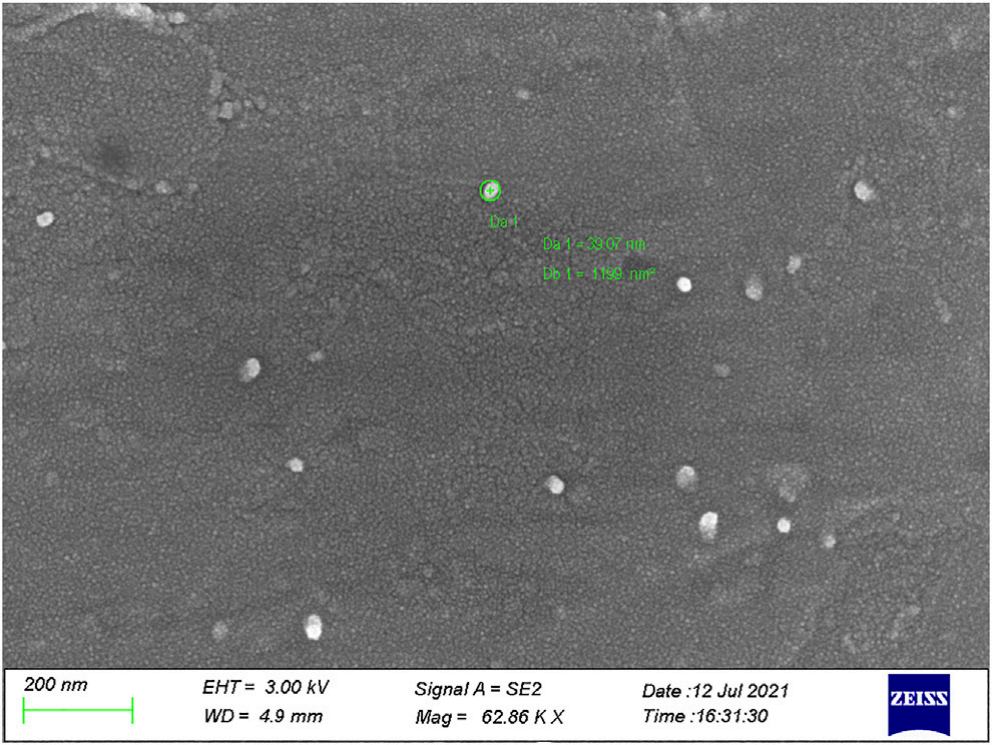
SEM image (at the 200 nm level) of the mixed crystal of the pyridine-modified DHB and 30 cP hydroxyl silicone oil.

On the contrary, mixing DHB with pyridine obviously changed the shape of the mixed crystal of matrix and analyte. As shown in Figure 6, the crystal structure almost disappeared, and the image was filled with kinds of crystal particles. The large particles had the diameters of only 39 nm. In addition, there were much more particles with the diameters less than 10 nm, which is almost near the size of a molecule. Similarly, the addition of NaAc also did not obviously change the shape of the mixed crystal of matrix and analyte, in which many scattered crystal particles had diameters of 38 nm and much more particles showed diameters less than 10 nm (Supplementary Figure S5). The above experimental results showed that the mixture of hydroxyl silicone oil in the pyridine-modified DHB matrix was more uniform, in which the crystal cluster diameters decreased and the solubility increased obviously. As a result, it is much easier for the matrix to transfer the absorbed laser energy to the analyte in the process of ionization. Thereby, much better ionization efficiency was obtained for hydroxyl silicone oil, when using pyridine-modified DHB as the MALDI-TOF matrix.

### Characterization of Oligomeric Hydroxyl Silicone Oils

**FIGURE 7:**
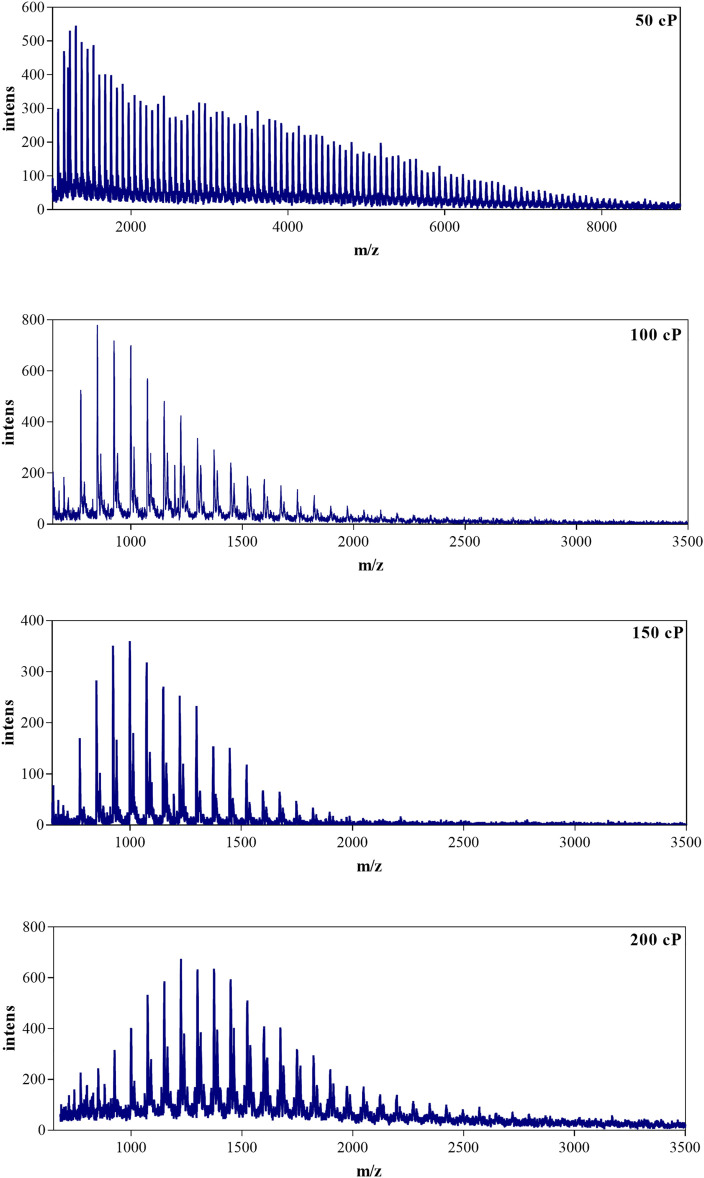
MALDI-TOF MS of hydroxyl silicone oils (50 cP, 100cP, 150cP and 200 cP) with the matrix of DHB modified by pyridine and NaAc.

According to the optimized experimental parameters, various oligomeric hydroxyl silicone oils were characterized by MALDI-TOF (Figure 2C and Figure 7). As can be seen, the *m/z* ratio of 30 cP hydroxyl silicone oil mainly ranges from 1,000 to 7,000, and the MS data of the typical 30 cP hydroxyl silicone oil are listed in Table 1. The mass gap (74 Da) of the neighboring peaks in the MS indicates the repeating unit of (SiOMe_2_). The identity of the attached Na^+^ can give a reasonable ascription of all the signal in the MS of the hydroxyl silicone oil, which agrees well with the fact that it tends to be ionized by the attachment with Na^+^.

**TABLE 1:** MALDI-TOF MS data of 30 cP hydroxyl silicone oil.

[M + Na]^+^	Chemical formula	Intensity	(M + Na)^+^	Chemical formula	Intensity
*m/z*			*m/z*		
1,078	C28H86O15Si14Na	124	2,710	C72H218O37Si36Na	291
1,153	C30H92O16Si15Na	203	2,784	C74H224O38Si37Na	268
1,227	C32H98O17Si16Na	266	2,858	C76H230O39Si38Na	259
1,301	C34H104O18Si17Na	324	2,933	C78H236O40Si39Na	237
1,375	C36H110O19Si18Na	329	3,007	C80H242O41Si40Na	242
1,449	C38H116O20Si19Na	369	3,081	C82H248O42Si41Na	221
1,523	C40H122O21Si20Na	374	3,155	C84H254O43Si42Na	189
1,597	C42H128O22Si21Na	377	3,229	C86H260O44Si43Na	220
1,671	C44H134O23Si22Na	376	3,303	C88H266O45Si44Na	189
1745	C46H140O24Si23Na	399	3,377	C90H272O46Si45Na	167
1819	C48H146O25Si24Na	400	3,451	C92H278O47Si46Na	161
1894	C50H152O26Si25Na	400	3,526	C94H284O48Si47Na	151
1968	C52H158O27Si26Na	404	3,600	C96H290O49Si48Na	149
2043	C54H164O28Si27Na	407	3,674	C98H296O50Si49Na	129
2,117	C56H170O29Si28Na	394	3,748	C100H302O51Si50Na	148
2,191	C58H176O30Si29Na	406	3,822	C102H308O52Si51Na	129
2,265	C60H182O31Si30Na	389	3,896	C104H314O53Si52Na	130
2,339	C62H188O32Si31Na	383	3,970	C106H320O54Si53Na	130
2,414	C64H194O33Si32Na	351	4,045	C108H326O55Si54Na	99
2,488	C66H200O34Si33Na	351	4,119	C110H332O56Si55Na	122
2,562	C68H206O35Si34Na	338	4,193	C112H338O57Si56Na	111
2,636	C70H212O36Si35Na	299	4,267	C114H344O58Si57Na	95

Thus, the number-average molecular weight (*M*_*n*_), weight-average molecular weight (*M*_*w*_), dispersity (*PD*), and hydroxyl content of silicone oils (Si-OH%) were calculated to be 2,276, 2,553, 1.12, and 1.68, respectively, according to the following formula:

$$M_{n}=\sum (n_{i}\times M_{i})/\sum n_{i},M_{w}=\sum (n_{i}\times M_{i}^{2})/\sum (n_{i}\times M_{i}),$$

$$PD=M_{w}/M_{n},OH\%=\sum ((n_{i}/\sum n_{i})/\times 34/M_{i})\times 100\%,$$

Here, *n*_*i*_ and *M*_*i*_ refer to the MS intensity and molecular weight of any component *i* of the oligomer.

50 cP hydroxyl silicone oil has the same mass gap (74 Da) of the neighboring peaks in the MS, but it shows a different mass distribution with a wider mass range (1,000–9,000 Da). As shown in Table 2, 50 cP hydroxyl silicone oil has a higher molecular weight, more dispersity, and less hydroxyl content.

**TABLE 2:** *M*_*n*_, *M*_*w*_, and *PD* of silicone oils with different viscosities.

Sample/viscosity	*M*_*n*_	*M*_*w*_	*PD*	Si-OH%
30 cP	2,383	2,658	1.12	1.61
50 cP	3,185	3,924	1.23	1.38
100 cP	1,135	1,215	1.07	3.19
150 cP	1,151	1,201	1.04	2.90
200 cP	1,460	1,554	1.06	2.48

As displayed in Figure 7, there are two series of peaks in the MS of 100, 150, and 200 cP silicone oils. The mass gap for the adjacent peaks is also 74 Da (SiOMe_2_) in each series of MS peaks. The main series of equidistant peaks is 16 Da less in molecular weight than the corresponding minor series of equidistant peaks, indicating that ionization of hydroxyl silicone oil by the attachment with Na^+^ results in the main one in the MALDI-TOF MS, and attachment with K^+^ results in the minor one. K^+^ originates from the residue catalyst (KOH) in the polymerization process. Also, the corresponding parameters of their main sequence peaks mass distribution are listed in Table 2.

Similarly, *M*_*n*_, *M*_*w*_, *PD*, and Si-OH% of several oligomeric hydroxyl silicone oils were also calculated and are summarized in Table 2. As can be seen, the hydroxyl silicone oils of 100 cP, 150 cP, and 200 cP have relatively higher viscosity than 30 cP and 50 cP, but they show much lower molecular weight (∼1,000 Da vs. ∼ 3,000 Da). Thus, molecular weight is not the deciding factor for the viscosity of the oligomeric hydroxyl silicone oil. The results indicate that the content of the silicon hydroxyl group, which results in the formation of an intermolecular hydrogen bond, exerts more influences on their viscosity (Table 2).

## Conclusion

In this work, the hydroxyl silicone oils have been successfully characterized by MALDI-TOF MS. The effects of the addition of pyridine and cationic reagent into matrix on the characterization of silicone oil were investigated. The results showed that the addition of pyridine and NaAc was beneficial to MALDI-TOF MS detection of hydroxyl silicone oils. The reduced baseline, the increased S/N, and a beautiful peak shape were obtained. Furthermore, the mixed crystal of matrix and 30 cP hydroxyl silicone oil was subjected to SEM and EDS analysis, and the results verified that addition with pyridine promotes the homogeneity of the crystal of DHB and silicone oil. Finally, several oligomeric hydroxyl silicone oils were characterized by MALDI-TOF MS, and the corresponding molecular weight and degree of polymerization were calculated, and the results indicated that the content of the silicon hydroxyl group, rather than the molecular weight, exerts obvious influences on their viscosity.

## Data Availability

The original contributions presented in the study are included in the article/Supplementary Materials, further inquiries can be directed to the corresponding author KJ, jiangkezhi@hznu.edu.cn.
