# Supplementary figures and images for: Characterizing Oligomeric Hydroxyl Silicon Oils by MALDI-TOF MS With the Pyridine-Modified Matrix
Source: Front Chem. 2021 Nov 23;9:755174. doi: 10.3389/fchem.2021.755174 (PMC8650622; doi:10.3389/fchem.2021.755174)

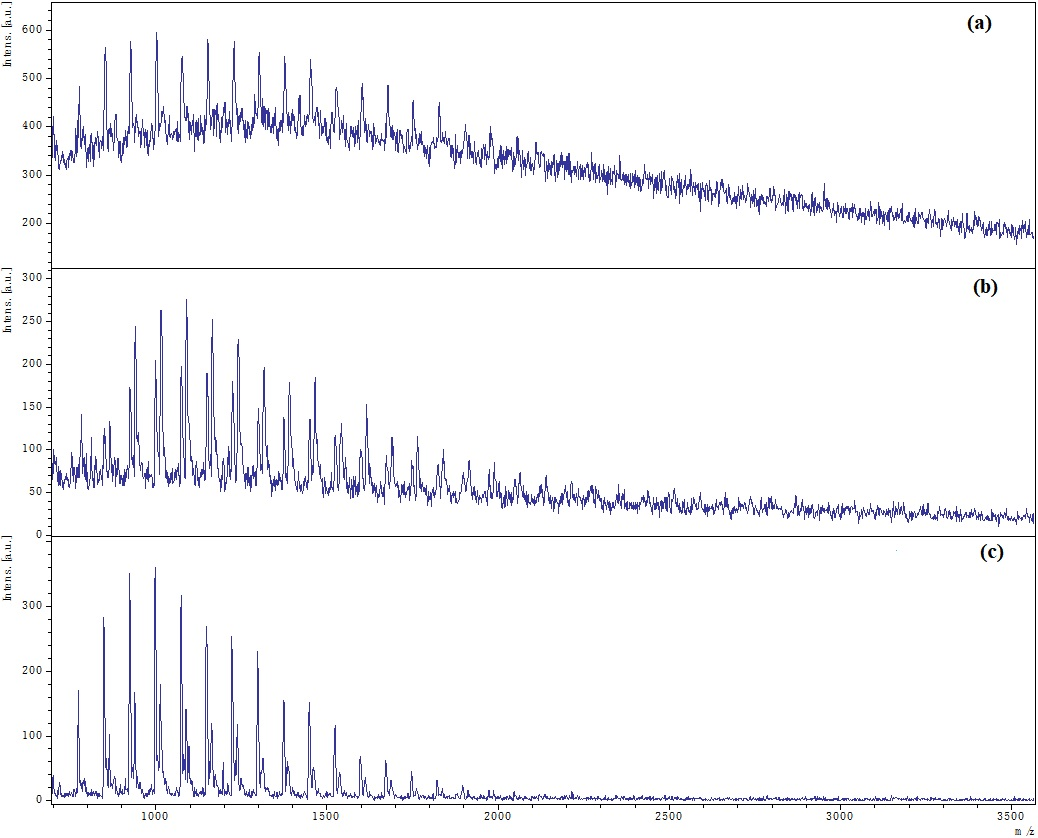

Supplement: Supplementary file 1 [file Image3.TIF]

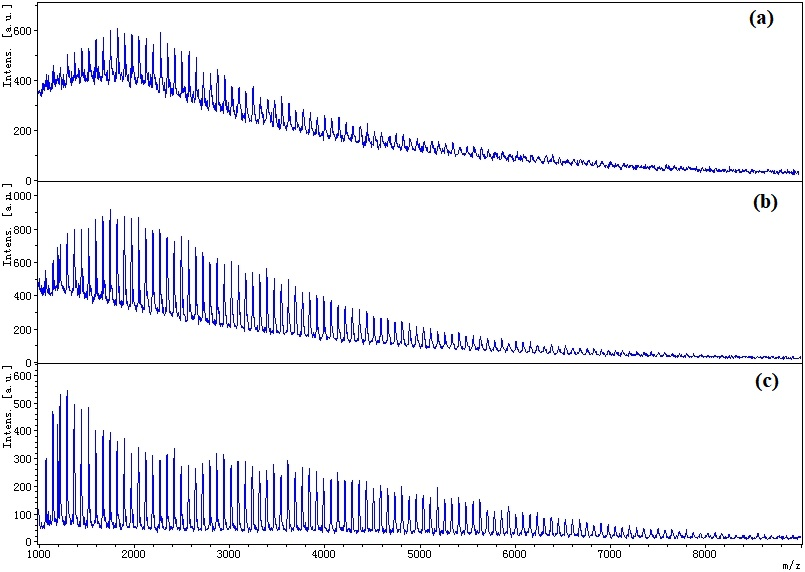

Supplement: Supplementary file 3 [file Image2.TIF]

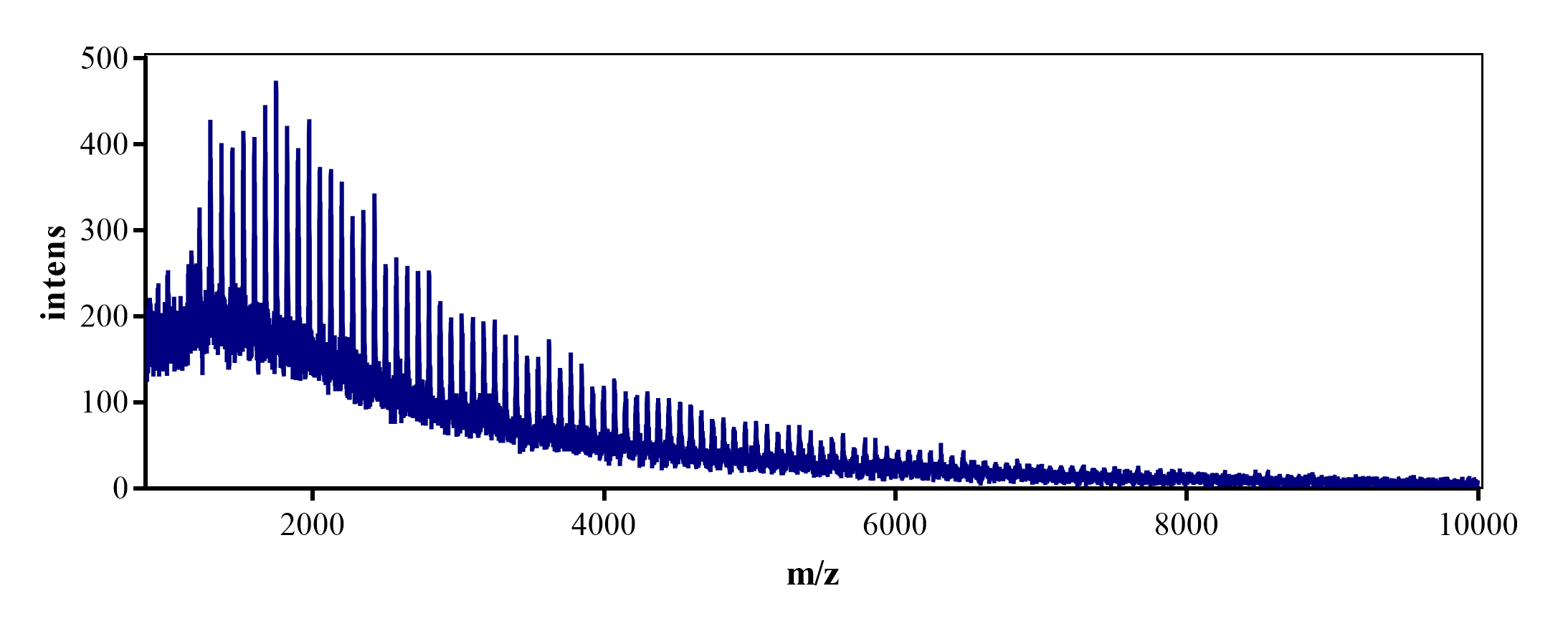

Supplement: Supplementary file 4 [file Image1.TIF]
